# Expression of sialic acids and other nonulosonic acids in *Leptospira*

**DOI:** 10.1186/1471-2180-12-161

**Published:** 2012-08-01

**Authors:** Jessica Ricaldi N, Michael A Matthias, Joseph M Vinetz, Amanda L Lewis

**Affiliations:** 1 Department of Medicine, Division of Infectious Diseases, University of California San Diego, School of Medicine, La Jolla, CA, 92093, USA; 2 Department of Pediatrics, University of California San Diego, School of Medicine, La Jolla, CA, 92093, USA; 3 Present address: Instituto de Medicina Tropical Alexander von Humboldt, Universidad Peruana Cayetano Heredia, Lima, Peru; 4 Present address: Department of Molecular Microbiology, Washington University School of Medicine, St. Louis, MO, 63110, USA

**Keywords:** Sialic acid, Nonulosonic acid, *Leptospira*

## Abstract

**Background:**

Sialic acids are negatively charged nine carbon backbone sugars expressed on mammalian cell surfaces. Sialic acids are part of a larger family of nonulosonic acid (NulO) molecules that includes pseudaminic and legionaminic acids. Microbial expression of sialic acids and other nonulosonic acids has been shown to contribute to host-microbe interactions in a variety of contexts, including participation in colonization, immune subversion, and behaviors such as biofilm formation, autoagglutination and motility. Previous research has suggested that some spirochetes may also express these molecules.

**Results:**

Here we use a combination of molecular tools to investigate the presence of NulO biosynthetic gene clusters among clinical and saprophytic isolates of the genus *Leptospira*. Polymerase chain reaction and Southern blotting suggested that a variety of leptospires encoded NulO biosynthetic pathways. High performance liquid chromatography and mass spectrometry analyses provided biochemical evidence that di-N-acetylated NulO molecules are expressed at relatively high levels by *L. interrogans* serovar Lai strain 55601, and at lower levels by *L. alexanderi* serovar Manhao and *L. fainei* serovar Hurstbridge. Endogenous expression of N-acetylneuraminic acid (Neu5Ac, the most common sialic acid) was documented in *L. interrogans* serovar Copenhageni strain L1-130. Neu5Ac biosynthesis is also supported by a unique gene fusion event resulting in an enzyme with an N-terminal N-acetylneuraminic acid synthase domain and a C-terminal phosphatase domain. This gene fusion suggests that *L. interrogans* uses a Neu5Ac biosynthetic pathway more similar to animals than to other bacteria. Analysis of the composition and phylogeny of putative NulO biosynthetic gene clusters in *L. interrogans* serovar Lai and serovar Copenhageni revealed that both strains have complete biosynthetic pathways for legionamimic acid synthesis, a molecule with the same stereochemistry as sialic acid. Lectin-based affinity purification of NulO-modified molecules, followed by mass spectrometric identification suggests post-translational modification of surface lipoproteins, including Loa22.

**Conclusions:**

*Leptospira* species encode NulO biosynthetic pathways and synthesize multiple NulO molecules including sialic acid. Additional studies are needed to clarify the exact context and functional significance of NulO expression. These findings have implications for immune evasion during systemic leptospirosis.

## Background

Leptospirosis, the most common zoonotic illness affecting humans, is caused by spirochetes of the genus *Leptospira*[1,2]. Some *Leptospira* species live exclusively in water or soil, while others cycle between environmental and mammalian reservoirs. *Leptospira* can colonize/infect renal tubules of a wide variety of wild and domesticated mammals. Human disease follows exposure to water or soil contaminated with urine of infected animals. Leptospirosis can be asymptomatic, or manifest as a mild flu-like illness. In another subset of individuals (5-10 % of patients) *Leptospira* can produce more serious systemic infections resulting in pulmonary hemorrhage, jaundice, renal failure, refractory shock, myocarditis, and/or aseptic meningitis.

Despite its medical importance, few virulence determinants of pathogenic *Leptospira* have been characterized in any detail. Investigation of the organism is hampered by its fastidiousness, slow growth in culture and the lack of available genetic tools. To date, only Omp-A like lipoprotein Loa22 has been demonstrated to be necessary for virulence, appearing to be cytotoxic and capable of inducing apoptosis. [3-5] LipL32, a major outer membrane protein of pathogenic *Leptospira*, is expressed *in vivo* and, although it has been shown to bind to host extra-cellular membrane, LipL32 does not seem to be required for acute or chronic infection *in vivo* in animal models. [6,7] Other potential virulence leptospiral factors include LigA and LigB that contain immunoglobulin-like repeats associated with adhesion to host cells in other gram-negative bacteria. Other proteins shown to have laminin binding activity *in-vitro* include LenA/LfhA/Lsf24 and related proteins LenBCDEF. LenA seems to also bind factor H of complement, so it might have more than one role in virulence. [8,9]. Leptospiral LPS, although not characterized in detail, has some unique characteristics which could explain why it is poorly recognized by the TLR4- MD2 complex. This diminished recognition could contribute to leptospiral survival in the bloodstream and dissemination. Other potential virulence factors for which more evidence remains to be published include mediators of motility and chemotaxis, including chemotaxis towards hemoglobin [10].

Sialic acids are a diverse family of acidic nine-carbon backbone (nonulosonic) monosaccharides found in abundance on the surfaces of mammalian cells and are sometimes expressed by microbial pathogens. The most common sialic acid in nature is N-acetylneuraminic acid (Neu5Ac). Expression of Neu5Ac by pathogenic bacteria has been linked mechanistically to complement and neutrophil evasion in disseminated infections with *Streptococcus* and *Neisseria* and with the induction of autoimmune neuropathy following infection with *Campylobacter*. Sialic acids are part of an even wider family of di-N-acetylated nonulosonic acid (NulO) sugars, which also includes pseudaminic and legionaminic acids. Legionaminic acid was first described as part of the *Legionella* lipopolysaccharide O-antigen [11], which is thought to have roles in environmental and host associations [12]. Legionaminic and pseudaminic acids are also found as post-translational modifications of flagellin, best studied in *Campylobacter* and *Helicobacter*[13,14]. Even further, recent data suggest that in *Helicobacter* proteins other than flagellins may also undergo glycosylation [15]. Our recent genomic and phylogenetic analyses indicated the presence of NulO biosynthetic gene clusters in the available genomes of *L. interrogans*[16]. In this study, we sought to investigate the presence of NulO biosynthetic gene clusters in other *Leptospira* species and to determine whether these genes produced functional biosynthetic pathways. Here we define the presence of putative nonulosonic acid biosynthetic gene clusters in a variety of *Leptospira* species. Further biochemical investigations show that some *Leptospira* are capable of endogenous synthesis of nonulosonic acids, including sialic acids.

## Results and discussion

### Nonulosonic acid biosynthetic gene clusters are present among pathogenic and some intermediately pathogenic *Leptospira* species

**Figure 1:**
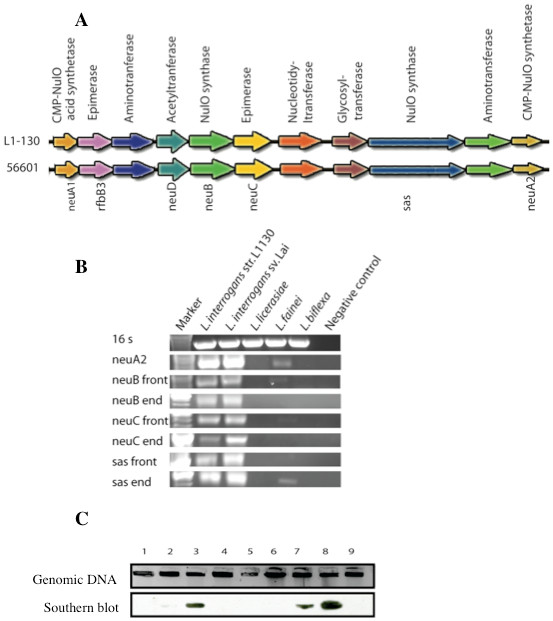
** *Leptospira* gene clusters predict nonulosonic acid biosynthesisA.** The sequenced genome of *L. interrogans* serovar Copenhageni L1-130 (top) and *L. interrogans* serovar Lai strain 56601 (bottom) encode a cluster of genes with predicted activities in the synthesis of sialic acids (N-acetylneuraminic acid) or related molecules. **B**. PCR of sialic acid cluster genes shows DNA amplification in pathogenic *Leptospira* species. Integrity of DNA was confirmed by amplification of the 16 S rRNA gene. **C**. Southern blots probed for the NeuA-2 region of the gene cluster using a DIG-labeled oligonucleotide. Genomic DNAs from the following bacteria were probed as described in materials and methods: 1) *S. enterica*, 2) *L. interrogans* serovar Lai strain 55601, 3) *L. interrogans* serovar Copenhageni strain L1-130, 4) *L*. *biflexa* serovar Patoc, 5) *L. licerasiae* (rat isolate CEH 008), 6) *L. licerasiae* isolate MMD4847), 7) *L. interrogans* serovar Icterohaemorrhagiae (isolate MMD 3731), 8) *L. fainei* serovar Hurstbridge, 9) *S. enterica*.

The genome sequences of *L. interrogans* serovar Copenhageni strain L1-130 and *L. interrogans* serovar Lai strain 55601 contain genes predicted to synthesize sialic acids or related molecules (Figure 1A). Using PCR and Southern blotting, we evaluated the presence of this gene cluster in other isolates of *Leptospira*, including pathogenic, saprophytic, and intermediate strains. Polymerase chain reactions using primers designed from the genome strains amplified genes in the pathogenic strains *L. interrogans* serovar Copenhageni and Lai but not in the saprophyte *L. biflexa* (Figure 1B)*.* Interestingly, one of the intermediate strains, *L. licerasiae*, gave a negative result, while the other, *L. fainei*, gave a faint positive. Control reactions using primers designed from 16S rRNA gene showed amplification in all the samples, verifying DNA integrity. A probe based on the *neuA2* gene of *L. interrogans* was used for southern blotting of genomic DNA from a number of *Leptospira* reference strains and isolates. These experiments confirm and extend the PCR data. Of particular interest is a pair of wild rodent isolates of *Leptospira* in lanes 6 and 7 (MMD4847 identifies as *L. licerasiae* and MMD3731 identified as *L. interrogans* serovar Copenhageni). Whereas the intermediately pathogenic *L. licerasiae* strain did not give a positive result, the pathogenic serovar Copenhageni isolate gave a strong positive band. Also, the intermediate strain *L. fainei* gave a positive result in southern blotting, further confirming the faint positive result observed by PCR for this strain. Since low sequence identity between primers or probes and the target sequences from less closely related species could produce a negative result in these experiments, other more functional assays were utilized next.

### DMB-derivatization and HPLC-MS analysis reveals multiple varieties of nonulosonic acids expressed by *Leptospira*

**Figure 2:**
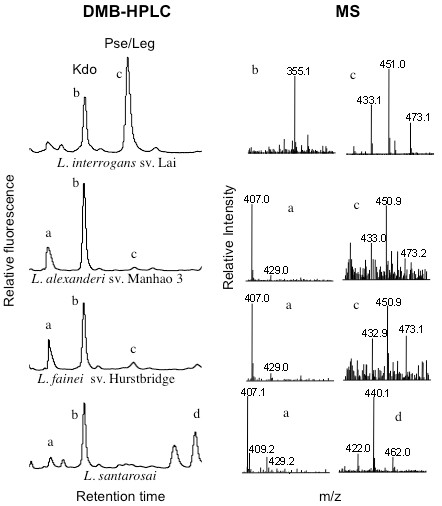
** *Leptospira* express mainly di- *N* -acetylated nonulosonic acids.** Nonulosonic acids were released from *Leptospira* isolates and fluorescently derivatized with DMB followed by HPLC as described in Materials and Methods. Selected peaks were subjected to electrospray ionization mass spectrometry. Pse and Leg refer to the di-N-acetylated nonulosonic acids pseudaminic and legionaminic acids, closely related isomers with an identical DMB-derivatized mass of 451. Kdo is a related eight-carbon backbone monosaccharide common to the core region of lipopolysaccharide. All MS data are shown from 400–500 m/z, except for representative MS data shown for peak b (Kdo), shown from 300–400 m/z. Each of these strains was analyzed in 2–3 independent experiments with similar results.

Strains were evaluated biochemically to determine whether nonulosonic acid biosynthetic pathways were functional in different species and strains of *Leptospira*. Bacteria were hydrolyzed with mild acetic acid to release nonulosonic acid species, and low molecular weight fractions were fluorescently derivatized with 1,2-diamino-4,5-methylene dioxybenzene (DMB), a molecule that specifically reacts with alpha keto acids, including NulOs. DMB-derivatized reaction products were separated by high performance liquid chromatography (HPLC) with a tandem electrospray ionization mass spectrometer. As expected by the Gram-negative-like structure of *Leptospira*, all samples displayed an early-eluting HPLC peak corresponding to the retention time and mass of 2-keto-3-deoxy-D-manno-octulosonic acid (Kdo). Kdo is an 8-carbon α-keto acid present in the core region of lipopolysaccharide in most Gram-negative bacteria. It serves as an internal positive control in these assays (Figure 2 peak b, m/z 355) and allowed comparison between different HPLC runs. Masses of some DMB-derivatized peaks did not readily correspond to masses of known varieties of nonulosonic acids (for example Figure 2 peak a, 407 and peak d, 440). It is not known whether these masses represent nonulosonic acids. In contrast, a consistent m/z of 433 (peak c) indicates the presence of di-N-acetylated nonulosonic acids and was found in pathogenic *L. interrogans* serovar Lai and *L. alexanderi*, and intermediate strain *L. fainei*. In all cases, the DMB-derivatized di-N-acetylated masses were accompanied with characteristic masses corresponding to the hydrated and hydrated sodium salt (m/z 451 and 473 respectively). These biochemical data show that pathogenic and intermediately pathogenic strains of *Leptospira* are capable of expressing di-N-acetylated nonulosonic acids. However, in contrast, the pathogenic strain *L. santarosai* was not found to synthesize identifiable nonulosonic acid species at detectable levels (Figure 2). We also performed analyses on *L. biflexa* serovar Patoc. In this case, we observed the presence of Kdo by HPLC and mass spectrometry, but identifiable NulO molecules were not present at detectable levels (not shown).

**Figure 3:**
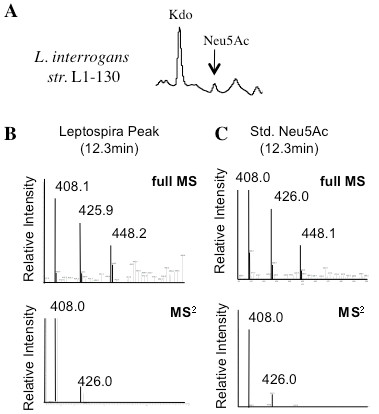
** *Leptospira interrogans* genome strain expresses sialic acid (Neu5Ac).** HPLC analysis demonstrates peaks consistent with Kdo and Neu5Ac in *Leptospira interrogans* str. L1-130. Confirmation of the L1-130 Neu5Ac peak assignment was performed by parallel derivatization and LCMS analysis of Neu5Ac (Sigma). The structure of DMB-derivatized Neu5Ac has a protonated exact mass (m+H) of 426.1. The dehydrated and sodium adduct have expected m+H values of 408.1 and 448.1 respectively. This experiment was performed twice with similar results.

**Figure 4:**
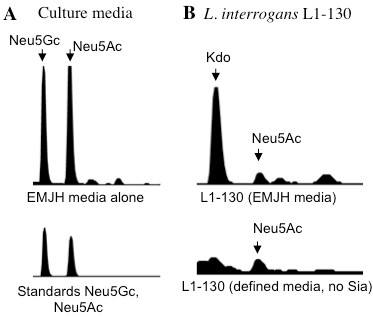
** *Leptospira interrogans* endogenously expresses N-acetylneuraminic acid (Neu5Ac).***L. interrogans* was grown in EMJH medium or in a chemically defined medium containing no exogenous sialic acid (this was confirmed by HPLC, not shown). Covalently bound Sias were released by mild acid hydrolysis and analyzed by DMB-derivatization and HPLC as described in previous figures and Materials and Methods. This experiment was performed twice with similar results.

Interestingly, HPLC analysis of the two different genome strains of *L. interrogans* (serovar Copenhageni strain L1-130 and serovar Lai strain 56601) gave distinct results. While *L. interrogans* serovar Lai expresses di-*N*-acetylated nonulosonic acid (Figure 2, m/z 433), strain L1-130 (serovar Copenhagenii) exhibited a peak with mass and retention time consistent with Neu5Ac (m/z 408, hydrated 426, and hydrated sodium salt 448) (Figure 3A-B). Additional MS^2^ analysis consistently reduced this trio of masses almost exclusively to the parent mass of 408 (Figure 3B), as expected based on the behavior of standard Neu5Ac derivatized in parallel (Figure 3C). Since the common animal sialic acids Neu5Ac and Neu5Gc were found in the standard culture media used for *Leptospira* (EMJH, Figure 4A), experiments were designed to exclude the possibility that *L. interrogans* strain L1-130 may incorporate exogenous sialic acid from the culture media. Unfortunately, the lack of a readily available genetic system for *Leptospira* rules out gene deletion as an approach to demonstrate endogenous synthesis. However, leptospires grown in defined serum-free media without sialic acids (as confirmed by HPLC) still produced a Neu5Ac peak, confirming that *L. interrogans* strain L1-130 synthesizes Neu5Ac and this sugar is not acquired from growth media (Figure 4B).

### Composition and phylogenetic analysis of NulO biosynthetic gene clusters and enzymes

**Figure 5:**
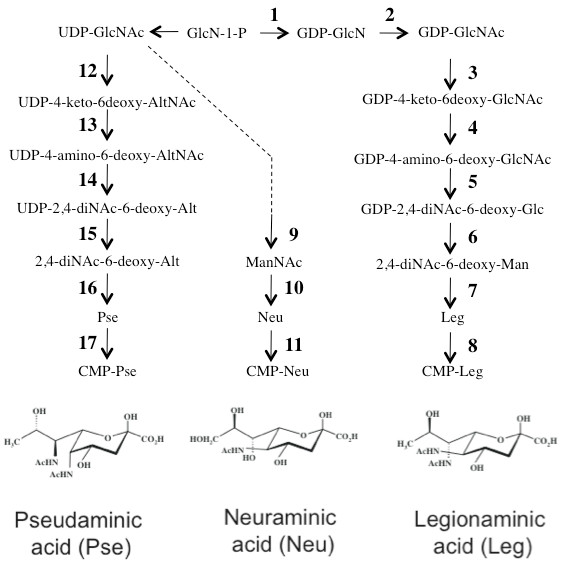
**Schematic of pseudaminic, legionamimic, and neuraminic acid biosynthetic pathways.** Studies of nonulosonic acid biosynthesis at the enzymatic level have been carried out with greatest resolution using *C. jejuni* and *H. pylori* as model systems [14,17-21,35]. Note that parallel arrows in this diagram indicate distinct, yet homologous enzymes. This schematic is based largely on the work of Schoenhofen et. al. Please refer to [14,18] and references within for more detailed descriptions of the enzymes and intermediates of these pathways.

Next we performed analysis of the composition and phylogeny of the putative NulO biosynthetic gene clusters and the enzymes they encode in *L. interrogans* serovars Lai (strain 56601) and Copenhageni (strain L1-130). Consistent with the biochemical analysis of *L. interrogans,* genomic analysis of the NulO gene cluster reveals that the organism encodes a complete pathway for di-N-acetylated nonulosonic acid biosynthesis (see Table 1 in comparison with Figure 5). There are multiple distinct open reading frames encoding synthesis of aminotransferases, NulO synthases, and CMP-NulO synthetases (see Table 1 and Figure 5), suggesting that *L. interrogans* may express multiple nonulosonic acid species, a conclusion supported by our biochemical investigations (Figure 2 and Figure 3).

**Table 1:** ** *L. interrogans* encodes a complete pathway for legionaminic acid synthesis**

** *Campylobacter* enzymes for legionaminic acid biosynthesis**[14,17-21]	** *C. jejuni* Pathway number (Figure 5)**	** *L. interrogans* L1-130 & 56601 NCBI accession numbers**	**Predicted *L. interrogans* Pathway number (Figure 5)**	**Predicted enzymatic Function**
PmtE (cj1329)	1	YP_002106	1	Glc-1-P guanyltransferase
		NP_711792		
GlmU	2	YP_000413	2	(housekeeping)
		NP_714003		N-acetyltransferase
LegB (cj 1319)	3	YP_002111	3	4,6-dehydratase
		NP_711787		
LegC (cj1320)	4	YP_002110	4	Aminotransferase in legionaminic acid synthesis (Figure 6A)
		NP_711788		
		YP_002103	4, 13, or ?	Aminotransferase
		NP_711795		
LegH (cj1298)	5	YP_002109	5	N-acetyltransferase
		NP_711789		
LegG (cj1328)	6	YP_002107	6	2-epimerase/NDP sugar hydrolase in legionamimic acid synthesis
		NP_711791		
LegI (cj1327)	7	YP_002108	7	Legionaminic acid synthase (Figure 6B)
		NP_711790		
		YP_002104	10	Legionaminic or neuraminic acid synthase (Figures 6B & 7)
		NP_711794		
LegF (cj1331)	8	YP_002102	8 or 11	CMP-Legionaminic acid or neuraminic acid synthetases (Figure 6C)
		NP_711796		
		YP_002112	8 or 11	
		NP_711786		

Phylogenetic comparisons were performed to provide additional insights into the potential functions of *Leptospira* nonulosonic acid biosynthesis enzymes. We included in the phylogenetic analysis the well-characterized enzymes of *Campylobacter jejuni* that participate in parallel pathways of legionamimic, pseudaminic, and neuraminic acid synthesis [14,17-21]. A schematic of these biosynthetic pathways is shown in Figure 5, noting the structural differences between neuraminic (sialic), legionamimic, and pseudaminic acids. These different NulOs are used by *C. jejuni* to modify a variety of different surface structures including the O-antigen of lipooligosaccharides, flagellin, and other surface proteins. To add further resolution to our phylogenetic analysis, we also included NulO biosynthetic enzymes from two *Photobacterium profundum* genome strains (3TCK and SS9), previously demonstrated to synthesize legionamimic and pseudaminic acids respectively [16]. In addition, homologous enzymes from other *Leptospira* genomes (*L. noguchii* str. 2006001870, *L. biflexa* serovar Patoc, *L. santarosai* str. 2000030832, *L. borgpetersenii* serovar Hardjo-bovis str. L550) were included in the phylogenetic analysis to better place the *L. interrogans* NulO enzymes into context with other putative leptospiral NulO biosynthetic enzymes.

**Figure 6:**
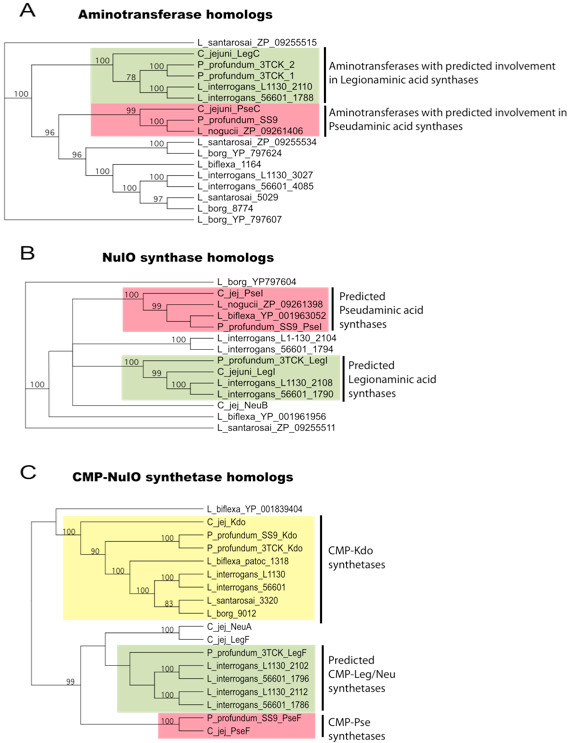
**Phylogenetic analysis of *L. interrogans* NulO biosynthetic enzymes.** Amino acid sequence alignments of “aminotransferase,” “NulO synthase,” and “CMP-NulO synthetase,” enzymes were performed using Clustal W and phylogenetic trees were built using the Neighbor-Joining method. *Campylobacter jejuni* enzymes with characterized functions in bacterial neuraminic, legionaminic, and pseudaminic acid biosynthesis [14,17-21] were compared to *L. interrogans* amino acid sequences encoded in the NulO biosynthetic gene cluster. Homologs of these enzymes from *P. profundum* strains 3TCK and SS9 were also included as they are know to synthesize legionamimic acid pseudaminic acids respectively [16]. Homologous enzymes from other selected *Leptospira* genomes (*L. noguchii* str. 2006001870, *L. biflexa* serovar Patoc, *L. santarosai* str. 2000030832, *L. borgpetersenii* serovar Hardjo-bovis L550) were also included in the phylogenetic analysis.

The phylogenetic analysis of *L. interrogans* NulO biosynthetic enzymes demonstrates that a subset of these enzymes is more closely related to the *C. jejuni* legionaminic acid biosynthetic enzymes and more distantly related to the pseudaminic acid biosynthetic enzymes (Figure 6). Specifically, the aminotransferases YP_002110 and NP_711788 and the NulO synthetases YP_002108 and NP_711790 in *L. interrogans* serovars Copenhageni and Lai respectively, are more closely related to legionaminic acid synthesis enzymes and more distantly related to *C. jejuni* and *P. profundum* pseudaminic acid synthesis enzymes (Figure 6A-B, note green and pink shading indicates legionaminic acid pseudaminic acid pathways respectively). A similar relationship was found for the predicted epimerase/NDP-sugar hydrolases YP_002107 and NP_711791(not shown). Moreover, we find that both homologs of the putative CMP-NulO synthetases in *L. interrogans* (YP_002102 and YP_002112 in L1-130 and NP_711786 and NP_711796 in 56601) are more closely related to legionaminic acid and neuraminic acid synthetases than to CMP-pseudaminic acid synthetases (Figure 6C). Note in this figure that CMP-Kdo synthases were included to provide contrast and distinguish between enzymes that likely participate in CMP activation of eight carbon sugars (i.e. Kdo) and nine carbon sugars (i.e. NulOs). The sequences retrieved by BLAST of these *Leptospira* genomes, together with their phylogeny, suggest that a number of leptospires do not encode homologs of CMP-NulO synthetases. In contrast, some leptospires encode putative NulO biosynthesis enzymes that are more closely related to the *C. jejuni* and *P. profundum* pseudaminic acid biosynthesis enzymes and more distantly related to the legionaminic acid enzymes (e.g. *L. noguchii* Figure 6A-B).

**Figure 7:**
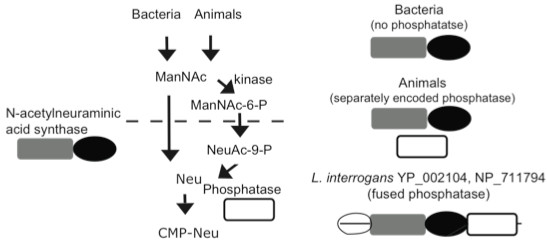
**C-terminal phosphatase domain fused to putative N-acetylneuraminic acid synthase suggests an animal-like Neu5Ac biosynthetic pathway in *L. interrogans* .**

In contrast to bacterial NulO biosynthetic pathways that synthesize Neu5Ac from ManNAc (N-acetyl mannosamine), the mammalian pathway relies on a NulO synthase with unique specificity for 6-phosphate-modified ManNAc, to generate 9-phosphate-modified Neu5Ac [22]. A set of adapter enzymes precede (kinase) and follow (phosphatase) the NulO synthase in the animal pathway (see Figure 7). In some cases, ‘adapter’ enzymes have become fused into the same open reading frame with one of the other nonulosonic acid biosynthesis genes. One example is the mammalian UDP-GlcNAc-2-epimerase, which is fused to a kinase that phosphorylates ManNAc to generate the substrate for the next step of the pathway, ManNAc-6-P. Interestingly, when performing analyses of *L. interrogans* NulO biosynthetic pathway, we noted that one of the NulO synthases encoded by *L. interrogans* (YP_002104 in serovar Copenhageni and NP_711794 in serovar Lai) has a unique C-terminal domain that is homologous to endonucleases that cleave phosphodiester bonds. By inference, we suggest that the route for N-acetylneuraminic acid biosynthesis in *L. interrogans* may be very similar to the animal pathway, condensing phosphoenolpyruvate with a phosphorylated 6-carbon intermediate to generate a phosphorylated 9-carbon sugar, followed by dephosphorylation catalyzed by the fused C-terminal phosphatase domain (Figure 7). This enzyme is distantly related to other NulO synthases and did not cluster with animal neuraminic acid synthases when these enzymes were included in the analysis (not shown), suggesting that this biosynthetic route may be ancestral. This conclusion is supported by previous evolutionary analyses of NulO pathways [16].

### Nonulosonic acids are elaborated on *Leptospira* surface lipoproteins

**Figure 8:**
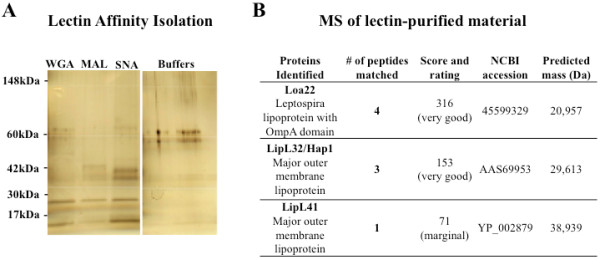
**Proteomic analysis suggests nonulosonic acids are present on surface lipoproteins in *L. interrogans* L1-130 A.** Silver-stained PAGE gel of affinity purified sialylated molecules from *L. interrogans* lysate using spin-columns with immobilized sialic acid-binding lectins WGA, MAL, or SNA. **B**. Results of proteomic analysis to identify proteins purified in **A**.

Finally, efforts were made to identify the type of molecule(s) modified with sialic acids in *L. interrogans* strain L1-130. Immobilized sialic acid-binding lectins from *Sambucus nigra* agglutinin (SNA) and *Maackia amurensis* lectin (MAL), which recognize sialic acids in α2-6 and α2-3-linked sialic acids respectively, were used to affinity purify sialic acid-modified molecules in lysates of the L1-130 strain. Wheat germ agglutinin (WGA) also recognizes sialic acids, but is less specific, and also recognizes N-acetylglucosamine residues. As a control, buffers used in the solid phase assay were analyzed in parallel lanes of the gel, revealing that the faint bands present at ~60 kDa were part of the supplied buffers and *not* specific for sialylated *L. interrogans* molecules. Silver staining after SDS-Page gel electrophoresis of the eluted material from the affinity columns shows clear bands at ~21 kDa and ~25 kDa that are present at similar intensities in the MAL and SNA lanes (Figure 8A). Other bands appear to be enriched by affinity purification using one or the other lectin. For example, a faint band at ~43 kDa is apparent in the material isolated by MAL, but not by SNA. Alternatively, bands at ~15, ~37, and ~41 kDa are much stronger in the SNA-purified sample. These finding suggests that *L. interrogans* may modify surface structures with both α2-3- and α2-6-linked nonulosonic acids (Figure 8A). However, future studies should further investigate the molecule(s) modified by nonulosonic acids in leptospires, as well as their exact context and importance.

The affinity-purified material was subjected to DMB-derivatization and HPLC analysis, which showed the Neu5Ac peak, but not the Kdo peak (data not shown), strongly suggesting that this material was free of LPS-components. This does *not* rule-out that LPS may be modified with NulOs, just that LPS was not present in this affinity-purified preparation. We performed mass spectrometry to identify protein components in the affinity-purified material. Three proteins were identified by mass spectrometry (Figure 8B): Loa22, LipL32, and LipL41, all of which have been described in previous publications as surface-exposed lipid-linked outer membrane proteins of *L. interrogans*[23-27]. Indeed, Loa22 and LipL31 are among the most abundant proteins expressed on the Leptospira cell surface [28]. Loa22 was identified with the highest number of peptide matches. Loa22 is an outer membrane protein encoded within all *Leptospira* genomes sequenced to date. It has been observed to be upregulated *in vivo*[27] and it is one of very few leptospiral proteins so far that has been shown to be necessary for virulence [3]. Additional studies are needed to define the precise context of NulO expression on *L. interrogans* and understand its potential significance in virulence.

## Conclusions

Based on a combination of experimentation and *in silico* genomic analysis, we have demonstrated the function of NulO biosynthetic gene clusters in pathogenic and intermediately pathogenic species of *Leptospira*, several of which are capable of synthesizing di-*N*-acetylated NulO species, as well as the true sialic acid, N-acetyneuraminic acid, a finding of considerable consequence for the leptospirosis field. This finding expands the number of important human pathogens that utilize endogenous biosynthetic pathways to elaborate surface structures containing sialic acids and related NulO molecules [16]. Sialic acids have proven roles in complement evasion, intracellular survival, and biofilm formation [29], and evidence is emerging that some human pathogens with Neu5Ac on their surfaces can engage sialic acid-binding receptors (Siglecs) on leukocyte cell surfaces, resulting in phagocytosis or dampening of bactericidal activities [30-32]. The roles of other NulO molecules such as legionaminic and pseudaminic acids are less well defined, but these molecules have been shown to play roles in behaviors such as autoagglutination, motility, and host colonization [33-37]. Curiously, disease caused by *L. interrogans* includes bacteremia and meningitis as components of the clinical disease spectrum, similar to the well-characterized Neu5Ac-expressing human bacterial pathogens Group B *StreptococcusNeisseria meningitidisE. coli* K1, and *Haemophilus influenzae*. As genetic tools and small animal infection systems for study of *Leptospira* are further refined, analysis of the contribution of NulO biosynthesis to the virulence of this neglected disease can be further elucidated.

## Methods

### Strains and culture conditions

Intermediately pathogenic strains *L. licerasiae* serovar Varillal strains MMD3731, MMD4847 and CEH008 (isolated from rodents in Peru), *L. fainei* serovar Hurstbridge strain BUT 6^T^ and the saprophyte *L. biflexa* serovar Patoc were used for these experiments. Pathogenic *Leptospira* used in this study included *L. interrogans* serovar Copenhageni strain L1-130, *L. interrogans* serovar Lai strain 55601, and *L. interrogans* serovar Icterohaemorrhagiae wild rodent isolate MMD 3731 that were passaged fewer than 5 times *in vitro* after re-isolation from hamster liver to maintain virulence. *L. santarosai* and *L. alexanderi* serovar Manhao were originally isolated from clinical cases of leptospirosis and now serve as reference laboratory strains. Generally, *Leptospira* were cultivated at 30°C in Ellinghausen-McCullough-Johnson-Harris (EMJH) medium (catalog #279510, Becton Dickinson, Sparks, Maryland). Chemically-defined, sialic acid-free medium, prepared as previously described and verified by HPLC to be sialic acid free, was used to cultivate *Leptospira* in experiments where the lack of exogenous sialic acids was a necessary condition [38].

### PCR of sialic acid cluster genes

Primers based on the genome of *L. interrogans* L1-130 were designed for the detection of genes in the sialic acid cluster as follows: sasfrontF (5′- TCC GGA AAT GCG AAT GAT G-3′), sasfrontR (5′- CAC CGG GCA AAA GAC TAA CCT - 3′), sasendF (5′- CGG ATA TAG CGG ACG ATG TAA - 3′), sasendR (5′- CGC CAA AAA GCC AAG GAA - 3′), neuA2F (5′- TGA AGC GGC AAA AAG AGC - 3′), neuA2R (5′- TGA AAT AAC ATG CCG ACA AAT A - 3′), neuCfrontF (5′- CGC TAC GGG AAT GCA TCT GTC TC - 3′), neuCfrontR (5′- CCC ATT CCC CCA ACC AAA AA - 3′), neuCendF (5′- GGC GAG GAT CCT TCT AAT GTT TTT - 3′) and neuCendR (5′- ACT CGC TCC GCC TTC ACC A - 3′). PCR reactions were prepared using 0.2 mM of each primer in a 20 *μ*L reaction with DNA from the pathogens *L. interrogans* Lai, *L. interrogans* L1-130, the intermediates *L. licerasiae* and *L. fainei* and the saprophyte *L. biflexa* serovar Patoc. NeuA2 and neuBfront reactions used an annealing temperature of 52°C. NeuCfront, neuCend, sasfront and sasend were run using an annealing temperature of 58°C. A 16 S gene PCR reaction using previously published primers fLIP and rLIP1 was used as a control for integrity of DNA.

### NeuA2 southern blot

Genomic DNA samples of *Salmonella enterica*, *L. interrogans* serovar Lai str. 56601, *L. interrogans* serovar Copenhageni str. L1-130, *L. biflexa* serovar Patoc, *L. licerasiae* strains CEH008 and MMD4847, *L. interrogans* serovar Icterohaemorrhagiae str.MMD3731 and *L. fainei* serovar Hurstbridge were prepared into plugs using 1 % agarose and 0.5x TBE. These were subjected to depurination and denaturing conditions. DNA was then transferred to a positively charged membrane via overnight capillary transfer with 20x SSC. Finally the DNA was cross-linked to the membrane using short wave DNA for 15 min. 10 mL of pre-hybridization solution (QuikHyb, Stratagene) were warmed to 40°C prior to hybridization. Hybridization was done overnight at 40-42°C using the same solution and adding 10 mL of DIG-labeled PCR product of primer neuA2F (5′ - TGA AGC GGC AAA AAG AGC - 3′) and neuA2R (5 ′- TGA AAT AAC ATG CCG ACA AAT A - 3′). 2xSSC at room temperature and 1x SSC at 68°C were used for stringency washes. A chemiluminescent substrate and an alkaline phosphatase conjugated anti-DIG antibody were used to demonstrate binding of the probe.

### Mild acid hydrolysis and DMB-derivatization of nonulosonic acids

Mild (2 N) acetic acid hydrolysis was performed to release surface nonulosonic acids from *Leptospira*. 4 N acetic acid was added to an equal volume of extensively washed and resuspended pellets followed by 3 h of incubation at 80°C. The resulting soluble fraction was filtered to remove large molecular weight components and derivatized with DMB (1,2-diamino-4,5-methylene dioxybenzene), a reagent that reacts with the α-keto acid portion of nonulosonic acids. Final reaction conditions were 7 mM DMB, 18 mM sodium hydrosulfite, 1.4 M acetic acid, and 0.7 M 2-mercaptoethanol. Derivatization was carried out for 2 hours at 50°C in the dark.

### High performance liquid chromatography and mass spectrometry

DMB-NulO derivatives were resolved by HPLC using a reverse phase C18 column (Varian) eluted isocratically at a rate of 0.9 ml/min over 50 minutes using 85 % MQ-water, 7 % methanol, 8 % acetonitrile as previously described [16,39,40]. In some experiments, HPLC was performed without online mass spectrometry and detection of fluorescently labeled NulO sugars was achieved using an online fluorescence detection using excitation and emission wavelengths of 373 nm and 448 nm respectively. In other experiments HPLC was combined with online mass spectrometry using a Thermo-Finnigan model LCQ ion trap mass spectrometer system. When mass spectrometry was performed, the mobile phase also included 0.1 % formic acid, and online UV detection of DMB-NulO molecules preceded mass spectrometric analysis. We note that similar HPLC-MS analyses have been described previously DMB-derivatized α-keto acids [39-41].

### Phylogenetic analysis

We performed BLAST searches (blastp) against the NCBI genome database using as seeds the sequences of 1) proteins encoded by *Campylobacter jejuni* pseudaminic, legionaminic, and neuraminic acid biosynthetic pathways or 2) enzymes encoded in the *Leptospira interrogans* NulO biosynthetic gene cluster (Figure 1A). NCBI accession numbers are provided in Table 1 and a schematic of the biosynthetic pathways is illustrated in Figure 5. Complete protein sequences of homologous amino acids were aligned using ClustalW in MacVector 11.1.1 software and alignments were checked manually. The Neighbor Joining (NJ) method was utilized for phylogenetic tree construction using MacVector 11.1.1 software, including 1000 Bootstrap replications to obtain confidence values for branches of the NJ trees.

### Solid-phase lectin binding

Whole cell lysates were prepared using three cycles of freeze-thawing of PBS washed *L. interrogans* serovar Copenhageni strain L1-130. In order to probe the abundance and nature of the sialylated molecules on *L. interrogans,* these lysates were fractionated using a lectin-based solid phase assay (Q Proteome Sialic Acid kit, Qiagen) using three immobilized sialic acid binding lectins: wheat germ agglutinin (WGA), *Sambucus nigra* agglutinin (SNA), and *Maackia amurensis* lectin (MAL), according to manufacturer’s instructions. Molecules captured by each of these lectins were eluted according to the manufacturers instructions. then analyzed by SDS-PAGE followed by silver staining (SilverQuest Silver Staining Kit, Invitrogen).

### Mass spectrometry

To determine whether *L. interrogans* uses nonulosonic acids for post-translational modification of proteins, pooled affinity-purified material from above mentioned experiment was subjected to denaturation, reduction, and alkylation, followed by trypsin digestion and MS/MS analysis using a nano-flow LC- tandem mass spectrometer. Peptide mass fingerprints identified in the affinity-purified material were used to identify L. *interrogans* proteins by searching against the NCBInr bacterial genome database.

## Competing interests

The authors declare that they have no competing interests.

## Authors’ contributions

JNR, MAM, JMV and ALL conceived and designed experiments, JNR and ALL acquired data, JNR, MAM and ALL analyzed and interpreted data, JNR and ALL drafted manuscript, JNR, MAM, JMV, and ALL revised manuscript critically for important intellectual content. All authors read and approved the final manuscript.
